# Structural basis of peroxidase catalytic cycle of human Prdx6

**DOI:** 10.1038/s41598-020-74052-6

**Published:** 2020-10-15

**Authors:** Rimpy Kaur Chowhan, Hamidur Rahaman, Laishram Rajendrakumar Singh

**Affiliations:** 1 Dr. B.R. Ambedkar Center for Biomedical Research, University of Delhi, Delhi, 110007 India; 2 Department of Biotechnology, Manipur University, Imphal, 795003 India

**Keywords:** Biochemistry, Biophysics

## Abstract

Peroxiredoxin 6 (Prdx6) is a ubiquitously expressed antioxidant non-selenium glutathione peroxidase that is known to play a major role in various physiological and pathological processes. It belongs to the family of peroxidases (referred to as Peroxiredoxins, Prdx’s) that work independently of any prosthetic groups or co-factors, and instead utilize a peroxidatic thiol residue for peroxide reduction. Mammalian Prdx’s are classified according to the number of Cys implicated in their catalytic activity by the formation of either inter-molecular (typical 2-Cys, Prdx1–4) or intra-molecular (atypical 2-Cys, Prdx5) disulfide bond, or non-covalent interactions (1-Cys, Prdx6). The typical and atypical 2-Prdx’s have been identified to show decamer/dimer and monomer/dimer transition, respectively, upon oxidation of their peroxidatic cysteine. However, the alterations in the oligomeric status of Prdx6 as a function of peroxidatic thiol’s redox state are still ambiguous. While the crystal structure of recombinant human Prdx6 is resolved as a dimer, the solution structures are reported to have both monomers and dimers. In the present study, we have employed several spectroscopic and electrophoretic probes to discern the impact of change in the redox status of peroxidatic cysteine on conformation and oligomeric status of Prdx6. Our study indicates Prdx6′s peroxidase activity to be a redox-based conformation driven process which essentially involves monomer–dimer transition.

## Introduction

Peroxiredoxin6 (Prdx6) belongs to a family of antioxidant enzymes called peroxiredoxins which rescue cells from oxidative stress by hydrolysing peroxides and peroxidised macromolecules^1^.They have been reported to be involved in various pathological conditions like neurodegeneration, diabetes, acute and chronic lung injury, cancer, etc^1–5^. Prdx family includes six isoforms in mammals, five in *Sachharomyces cerevisiae* and *Drosophila melanogaster*, up to 10 in plants with several specific ones located in the chloroplasts. These Prdx’s have been classified on the basis of multiple characteristics, including: (i) Enzymatic mechanism and cysteine involved in catalytic cycle (the most commonly used): this classification gave rise to qualification “typical 2-cys” (human Prdx 1–4), “atypical 2-Cys” (human Prdx5) and “1-Cys” (human Prdx6) Prdx categories; (ii) Sequence diversity and the position of the conserved cysteine: this system classified Prdx in six distinct types called A, B, C, D, E, and F. In mammals, only three of these types (A, B, and D) are present. Type A Prdx include typical 2-cys Prdx, homologous to human Prdx2. Type-B Prdx’s include 1-Cys Prdx’s homologous to human Prdx6, while, type-D Prdx’s includes homologous proteins to human Prdx5.

While typical Prdx (Prdx1-4) and atypical 2-Cys Prdx (Prdx5) have been identified to show decamer/dimer and monomer/dimer transition, respectively, upon oxidation of their peroxidatic cysteine^8,9,33,35^, the impact of redox modulation on 1-Cys Prdx structure is yet not clear. It is to be noted here that with the exception of Prdx6 all mammalian Prdx’s have 2-cysteine residues (peroxidatic, C_P_ and resolving, C_R_) that forms inter-molecular disulphide bond and are responsible for preferential homo-dimeric arrangement in oxidised state. For detailed review on structural biochemistry of Prdx family refer Sharapov et al. 2014 and Wood et al. 2004.

Prdx6′s peroxidase activity is mainly derived from a catalytically active C_P_–Cys 47 residue, located at the N-terminal part of the helix α2 of βαβ motif within thioredoxin fold^6^. The redox status of this peroxidatic cysteine regulates switching on/off of Prdx6′s peroxidase activity such that the enzyme with reduced Cys is active, oxidised Cys is reversibly inactive and hyperoxidised Cys is irreversibly inactive^6^. Redox cycling of Prdx6 to propel its peroxidase catalysis is believed to comprise of three steps—(i) self-oxidation and concurrent reduction of the peroxide substrate, (ii) glutathionylation of oxidised Prdx6 after hetero-dimerizing with pi-form of glutathione-S-transferase (πGST), and (iii) regeneration of catalytically active reduced Prdx6 and release of oxidised glutathione^7^. However, our knowledge regarding Prdx6 structure in these different redox states (especially the physiologically active structure) is very limited.

Despite progress in the study of other Prdx’s, limited data are available in the literature about the structural differences and quaternary structures between the oxidised and reduced forms of Prdx6 and hence the structural basis of redox regulation has not been completely explored. To date, there has been no systematic effort to investigate the conformational differences of the oxidised and reduced forms of Prdx6. Crystallographic and other analytical approaches have been used to understand the oligomeric nature of the two forms as other Prdx family members have been reported to exhibit oligomers of different nature in oxidised and reduced forms^8,9^. Unfortunately, crystal structures obtained for human Prdx6 yielded largely ambiguous results^10,11^ and deficiencies are noticed in the crystals^7,12,13^(https://www.ftp.wwpdb.org/pub/pdb/validation_reports/pr/1prx/1prx_full_validation.pdfhttps://www.files.rcsb.org/pub/pdb/validation_reports/b6/5b6m/5b6m_full_validation.pdf). Other studies including mass analysis of human Prdx6 samples via size exclusion chromatography and light scattering measurements have also been performed^14–17^. However, the exact redox status of the samples used in these studies has not been known. In the present study, we have employed several spectroscopic and electrophoretic probes to discern the impact of change in the redox status of peroxidatic cysteine of Prdx6 on enzyme conformation and oligomeric status. We observed that peroxidatic cysteine’s redox status of Prdx6 is associated with structural transitions that indeed determine the quaternary structure of the Prdx6.

## Materials and methods

### Materials

Trizma Base, EDTA, Sodium Hydroxide, Sodium dodecyl sulphate, Ethidium bromide, Imidazole, Tween-20, Tween-80, Sodium chloride, Potassium chloride, Glycerol, Acrylamide, bis-acrylamide, Ammonium persulphate, TEMED, Glycine, β-mercaptoethanol, Hydrogen peroxide (H_2_O_2_), Dithiothreitol (DTT), Horseradish peroxidase (HRP), 5,5′-dithiobis(2-nitrobenzoate) (DTNB), Bovine carbonic anhydrase, and Alpha-lactalbumin were purchased from Sigma Aldrich Pvt. Ltd.

Luria Bertani Medium and Luria Bertani Agar (Difco), PBS tablets (Biobasic), Ni–NTA resin (Qiagen GmbH, Germany), DNA markers (GeneDireX Inc., USA), Precision dual colour Protein marker (Bio-Rad). Ampicillin and Kanamycin were purchased from MP Biomedicals.

Primary (Monoclonal Anti-Prdx6, clone 3A10-2A11 antibody produced in mouse, WH0009588M1) and secondary (Anti-mouse IgG (Fab Specific)-Peroxidase antibody produced in goat, A3682) to detect full length human Prdx6 protein was procured from Sigma-Aldrich.

### Bacterial strains, media and culture conditions

*E.coli* strains used in the study were DH5α, M15[pREP4]. All media (Luria Bertani (LB) agar and broth) used for bacterial cultivation was autoclaved prior to usage. The antibiotics kanamycin (50 µg/ml) and/ or ampicillin (100 µg/ml) as per requirement were added after autoclaving in agar plates (after cooling the media ~ 60 °C) and broth (before inoculation). The strains were grown in conical flasks at 37 °C with shaking at 150 rpm to increase aeration. For long term storage, bacteria were stored at − 80 °C in LB medium containing 25% glycerol.

### Human Prdx6 protein expression and purification

The plasmid construct containing gene for Wt human Prdx6 was purchased from ThermoFisher Scientific. They assembled synthetic gene for Wt human PRDX6 synthetic oligonucleotides and/or from PCR products. The fragment was inserted into expression vector pQE30-Xa. The plasmid DNA was purified from transformed bacteria (DH5α cells) and concentration determined by UV spectroscopy. The final construct was verified by sequencing. The sequence congruence within the insertion sites was 100%.

*E. coli* M15 [pREP4] cells carrying pQE30-Xa/human Prdx6 construct were grown at 37 °C in 1L LB media containing ampicillin (100 μg/ml) and kanamycin (50 μg/ml),. On reaching the OD_600_ of 0.4–0.6, recombinant protein production was induced by addition of 0.5 mM IPTG. After further incubation at 37 °C for 4 h, cells were harvested by centrifuging at 8000 rpm for 30 min. Bacterial cell pellets were resuspended in lysis buffer (20 mM Tris (pH 7.0), 50 mM NaCl, 10 mM Imidazole, 0.5 mg/ml lysozyme, 0.005 mg/ml RNaseA, 0.005 mg/ml DNase), kept on rocker at 4 °C for 1 h, and sonicated on ice for 12 pulses of 30 s with gaps of 30 s in between. The final lysates were centrifuged at 14,000 rpm for 40 min, and the respective supernatants after passing through a syringe filter of 0.22 μm were loaded on a Ni–NTA agarose column pre-equilibrated with binding buffer ((20 mM Tris (pH 7.0), 50 mM NaCl, 10 mM Imidazole) and incubated overnight at 4 °C. Next day, the unbound proteins were collected as flowthrough. The column was washed with 3 bed volumes of wash buffer (20 mM Tris (pH 7.0), 50 mM NaCl, 60 mM Imidazole,), followed by protein elution using elution buffer (20 mM Tris (pH 7.0), 50 mM NaCl, 500 mM Imidazole) . The bound protein was eluted in fractions of 1 ml. The protein-containing fractions were pooled, incubated for an hour with 5 μM EDTA (to remove any His-bound metal ions), dialyzed against dialysis buffer (20 mM Tris (pH 7.0), 50 mM NaCl), and stored at − 20 °C. The purified proteins were checked on SDS-PAGE followed by immunoblotting with respective antibodies, and quantitated by Bradford assay using BSA as standard. Since, human recombinant Prdx6 is expressed as a fusion protein with a series of six histidine residues, the molecular weight of the monomer is predicted to be approx. 26,200 Daltons.

### Sodium dodecyl sulfate polyacrylamide gel electrophoresis (SDS-PAGE)

Approximately, 5 ml of resolving gel (12% acrylamide, pH 8.8) and 2 ml of stacking gel (5% acrylamide, pH 6.8) were prepared for electrophoresis. The protein samples were mixed with sample loading buffer (reducing dye) in a ratio of 5:1 and kept at 94 °C for 5–10 min. The samples were loaded on the SDS gel and separated using Mini-Protean3 cell (Bio-Rad) by applying 90 V (constant voltage). A dual colour pre-stained Protein Ladder (10 to 250 kDa, Bio-Rad) was loaded as a standard for molecular mass. Following electrophoresis, the gel was either stained with Coomassie Brilliant Blue R-250 or used for western blot analysis^18,19^. For full length images of all SDS-PAGE gels and immunoblot, please see supplementary information.

### Cross-linking with formaldehyde

Reduced and oxidised Prdx6 at 0.5 μM concentration was dissolved in 1 M HEPES buffer (pH 7.0) and incubated for 3 h in the presence of 0.6% and 1.2% formaldehyde [Utility of formaldehyde cross-linking and mass spectrometry in the study of protein–protein interactions]. The reaction was stopped by adding 1 M Tris (pH 7.0) and the crosslinked products stabilized. The sample was then mixed with equal volumes of non-reducing sample loading buffer, and analyzed by SDS-PAGE under non-reducing conditions.

### Native PAGE

Native PAGE analysis of the reduced and oxidised Prdx6 was carried out by loading samples with an average of 20 μg (30 μl load volume) per well and run at 90 V constant voltage per gel. The stacking gel, resolving gel, and the running buffer were prepared in the same way as the SDS-PAGE, except that (a) no SDS was used, (b) samples were not heat denatured, and (C) not exposed to reducing agents^20^. The gels were stained with Coomasie Brilliant Blue R-250 for further analysis.

### Pre-reduction of Prdx6

Prdx proteins are commonly oxidized after prolonged exposure to air or if purified under non-reducing buffers (Nelson, 2011). To reduce the protein, 1/10 volume DTT from a 100 mM stock is added to the protein solution (i.e. for 1 ml protein, add 0.1 ml DTT). To ensure complete reduction, Prdx6 (< 10 mg/ml) was incubated overnight at 4 °C with at least a 20 fold molar excess of DTT. DTT is then removed by dialysing Prdx6 sample against 20 mM Tris–HCl buffer (pH 7.0) at 4 °C for 2hours. Various Prdx’s and Thioredoxins have been reported to remain fully reduced for up to 6hours on ice after removal of DTT. The concentration of reduced dialysed Prdx6 protein is estimated using ε_280_ = 22,400 M^−1^ cm^−1^ (molar extinction coefficient calculated via ExPASy-ProtParam web tool).

### DTNB assay

Protein sulfhydryl (-SH) group estimation was carried out as described by Ellman with some minor modifications. The denaturing conditions used in the protocol allow accessibility to all protein thiols. Firstly, 5 μl of 30 mM DTNB is mixed with 4 M GdmCl, 20 mM Tris–HCl, 0.5 mM EDTA, pH 8.0 to make up the final volume of 1 ml, and after 2–3 min, the change in A_412_ is monitored to determine spontaneous TNB production. To this mixture, add 10 μl of Prdx6 sample, and again measure absorbance at 412 nm. The background absorbance measured in first step is subtracted, and no. of reduced thiols are calculated using ε_412_ = 14,150 M^−1^ cm^−1^.

### HRP competitive assay

The rate constant for reaction of Prdx (7.5 μM) with H_2_O_2_ (10 μM) was determined by competition with HRP. The peroxidase is converted by H_2_O_2_ to compound I, which in the absence of a reducing substrate remains stable under these conditions (Nelson, 2011). Spectra were recorded using a Jasco V-660 UV/Visible spectrophotometer. The concentration of HRP was determined by measuring A_403_ (ε_403_ = 1.02 X 10^5^ M^-1^ cm^-1^), and the extent of conversion to compound I was monitored as a decrease in absorbance at 398 nm(the isobestic point between HRP oxidation products, HRP-I and HRP-II). Following equation is used to compute the percentage inhibition of HRP by reduced and oxidised Prdx6^21,22^.

$$Percentage\; inhibition\; of\;HRP = \frac{\Delta Amax - \Delta Aobs}{{\Delta Amax}} \times 100 \%$$

where ΔAmax and ΔAobs is the average change in HRP absorbance in the absence and presence of Prdx6, respectively.

### Mass spectrometry (MS)

Intact mass of reduced Prdx6 was determined by an ESI-based MS. 2 ml of pre-reduced Prdx6 protein(2 mg/ml) is desalted using dialysis against2L of 1 mM Tris–HCl buffer (pH 7.0) for 2 h. The positive ion mass spectra were acquired on an Applied Biosystems, MDS Sciex (Model 4800 plus MALDI TOF/TOF Analyser), to obtain the final average mass of target protein. Spectra of the protein were obtained in linear mode.

### Circular dichroism (CD) measurements

CD measurements of reduced and oxidised Prdx6 dissolved in 20 mM Tris–HCl buffer (pH 7.0)were made in a Jasco J-810 spectropolarimeter equipped with a Peltier-type temperature controller with six accumulations^23^. Prdx6 concentration used for the CD measurements was 0.4 g/l (16 µM). Cells of 0.1 and 1.0 cm path length were used for the measurements of the far- and near-UV spectra, respectively. Necessary blanks were subtracted. The CD instrument was routinely calibrated with D-10-camphorsulfonic acid^24^. Secondary structure estimation from the Far-UV CD spectra was calculated using Yang’s method (https://www.ncbi.nlm.nih.gov/pubmed/4366945). Each spectrum was repeated at least three times and the mean curve was plotted using Sigma Plot Software 10.0 from Systat Software, Inc., San Jose California, USA, www.systatsoftware.com.

### Trp fluorescence spectroscopy

Fluorescence spectra of reduced and oxidised Prdx6 (0.1 mg/ml) dissolved in 20 mM Tris–HCl buffer (pH 7.0) was measured using Cary Eclipse Fluorescence Spectrophotometer (Agilent Technologies) with both excitation and emission slits set at 10 nm in a 3 mm quartz cuvette. For fluorescence measurements, Prdx6 protein was excited at 295 nm, while the emission spectra were recorded from 300 to 600 nm. Each spectrum was repeated at least three times and the mean curve was plotted using SigmaPlot Software 10.0 from Systat Software, Inc., San Jose California, USA, www.systatsoftware.com.

### Heat-induced denaturation studies

Thermal denaturation studies of Prdx6 at different redox states was carried out in Jasco J-810 spectropolarimeter equipped with a Peltier-type temperature controller at a heating rate of 1 °C per minute^23,25^. This scan rate was to provide adequate time for equilibration. Each sample was heated from 20 to 85 °C. The change in ellipticity with increasing temperature was followed at 222 nm for 16 μM Prdx6. Measurements were repeated three times and the mean curve was plotted using Sigma Plot Software 10.0 from Systat Software, Inc., San Jose California, USA, www.systatsoftware.com^24^. Each heat-induced transition curve was analysed for Tm (midpoint of denaturation) using Origin 7.0 (OriginLab Corporation, Northampton, MA, USA, www.originlab.com)^26^.

### Dynamic light scattering (DLS)

Dynamic light scattering measurements for reduced and oxidised protein (1.0 mg/ml) were obtained using a Zetasizer Micro V/ZMV 2000 (Malvern, UK)^27^. For each sample, fifteen measurements (one measurement takes 30 s acquisition time) at sensitivity of 10% were made using an incident laser beam of 689 nm at a fixed angle of 90°^28^. Data analysis was done using Zetasizer software provided by Malvern to get hydrodynamic diameters (H_d_), and polydispersity which is a measure of the standard deviation of the size of the particles^27^.

### Size exclusion chromatography

Size-exclusion chromatography was performed by using Sephadex G-75 column. The sephadex powder swelling and gel chromatographic column (1 cm radius, 11 cm height, 35 ml bed volume) preparation was done as per manufacturer recommendations. 3 bed volumes of 20 mM Tris–HCl buffer (pH 7.0) were passed through the column to stabilise and equilibrate the gel bead. 500ul of Blue dextran (2 mg/ml), reduced Prdx6 (2 mg/ml) and oxidised Prdx6 (2 mg/ml) were eluted out of the column using 1 bed volume of 20 mM Tris–HCl buffer separately. Each eluted fraction (1 ml) was monitored for the presence of Blue dextran and protein using UV/Vis Spectroscopy at 540 nm and 280 nm respectively. Absorbance values were plotted against the elution volume to get the elution profile of blue dextran, reduced and oxidised Prdx6. The volume required to elute the blue dextran and protein at its maximum elution (peak position) is considered as the void volume and elution volume, respectively.

## Results

### Generation of oxidised and reduced human Prdx6

**Figure 1:**
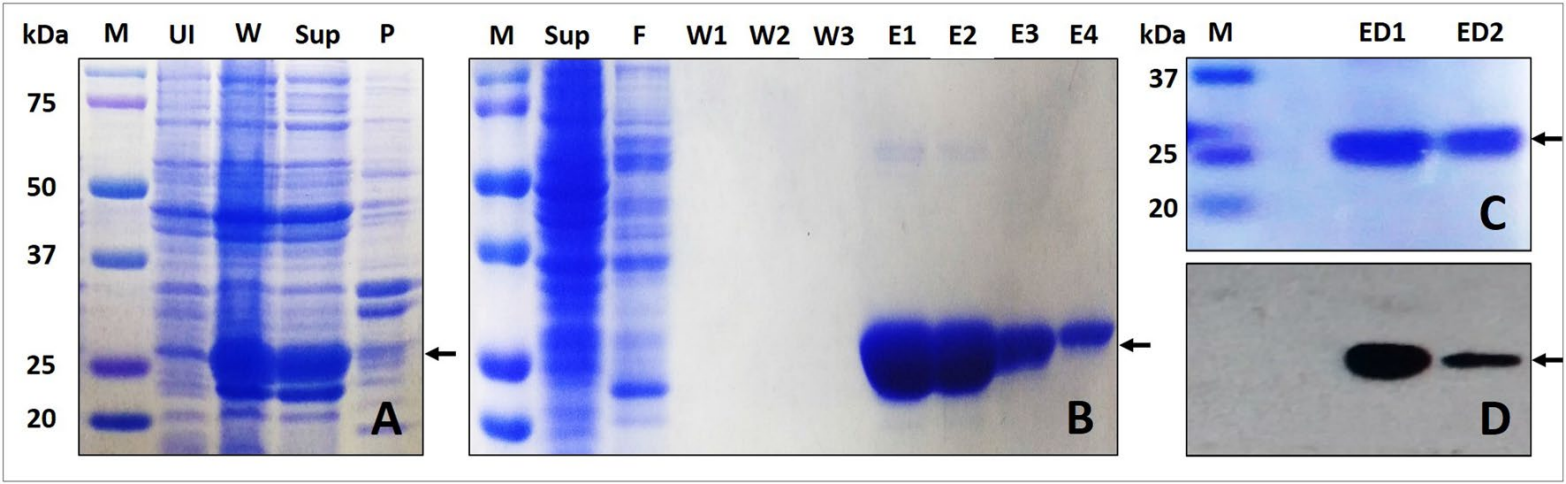
Expression and Purification of Prdx6. 12% SDS-PAGE showing (**A**) IPTG Induction of Prdx6 cDNA expression in E.coli M15[pREP4] cells containing pQE30-Xa_Prdx6-human vector, (**B**) Poly-His/Ni–NTA Affinity chromatography based purification profile of human Prdx6, and (**C**) Protein recovery after dialysis. (**D**) Immunoblot showing the presence and purity of desalted Wt human Prdx6 protein after incubating with anti-human Prdx6 antibody. The arrow indicates the position of the recombinant protein. M: Marker proteins (the molecular weight (kDa) is shown on the left); UI: Uninduced cells; W: Induced cells; Sup: Lysate from induced cells; P: Debris from induced cells; F: Flow-through; W(1–3): Washes; E(1–4): Elutions; ED (1–2): Elutions after desalting using dialysis.

For in vitro analysis, we have used human Prdx6 protein purified via an *E.coli* expression system. The purified protein identity is verified via immunoblotting of dialysed desalted protein with anti-human Prdx6 antibody (Fig. 1). The reduced protein is generated via overnight incubation with excessive amount of DTT (an established in vitro electron donor of Prdx6) followed by its removal via dialysis. On the other hand, the purified protein that has not yet been exposed to either an oxidising or reducing agent was assumed to have cysteine group in sulfenic acid form (Cys-SOH) and not in thiol (Cys-SH), disulphide (Cys-S–S-Cys), sulfinic (Cys-SO_2_H) or sulfonic form (Cys-SO_3_H) because—(a) human Prdx6 purified and crystallized under non-oxidising/non-reducing conditions are known to have a stable mono-oxidised peroxidatic cysteine^7,11^, (b) the distance of Cys 91 residue approximately 17 A° away from peroxidatic Cys prevents formation of an intra-molecular disulphide bond^11^, (c) localization of Cys 47 at the bottom of a narrow pocket by the first turn of the helix α2 does not allow formation of intermolecular disulphide bond^11^ ,and (d) no external peroxide substrate is added which is essential for hyperoxidation of Prdx6^5,29^. This assumption was further confirmed by estimating the thiol content of reduced and oxidised protein using DTNB Assay. It is seen in Table 1 that the oxidised Prdx6 exhibits no reactivity to DTNB. We also observed that more than 90% of Prdx6 population remained in the reduced state at 6 h after DTT removal, beyond which the thiol group started oxidising by environmental oxygen at a relatively faster rate (data not shown).

**Table 1:** Quantification of Total Thiol Content in the Prdx6 Samples Using Absorption Spectroscopy and DTNB Assay.

	Absorption spectroscopy [Protein]^a^ ([expected thiol]^b^) (μM)	DTNB assay (Total thiol) (μM)
Reduced Prdx6	20 (40)	37 ± 1.2
Oxidised Prdx6	20 (40)	0.5 ± 0.2

^a^As measured photometrically at 280 nm using the extinction coefficient (ε_280_ = 2.24 × 10^5^ M^−1^ cm^−1^).

^b^As calculated by multiplying the protein concentration with no. of Cys residues in the protein (Human Prdx6 has 2 Cys residue).

### Oxidation of Prdx6′s peroxidatic cysteine reduces its peroxidase activity

**Figure 2:**
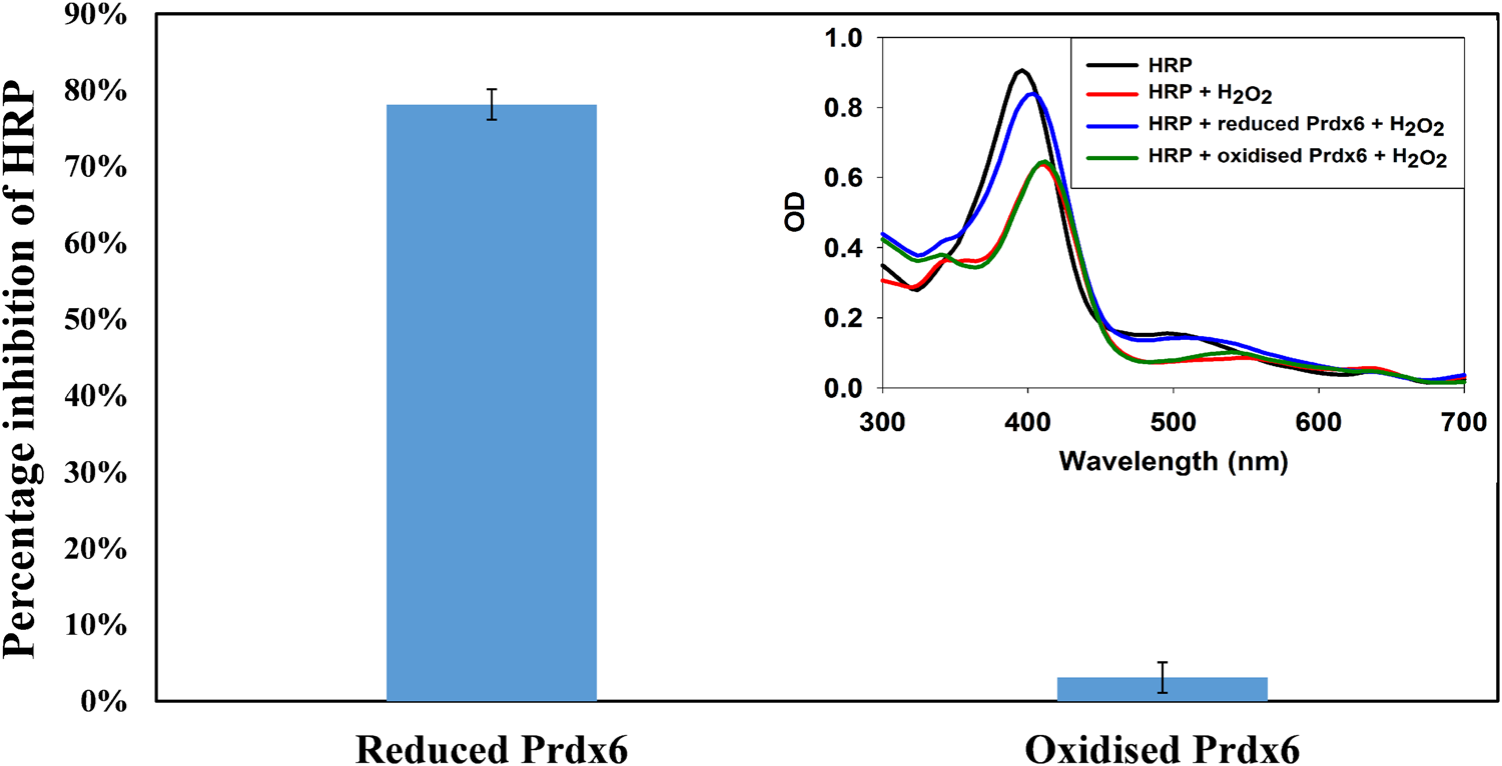
Determination of Peroxidase Activity of Prdx6 Using HRP competitive assay. The plot of percentage inhibition of HRP oxidation by hydrogen peroxide in the presence of reduced and oxidised human Prdx6 as an indicative of the peroxidase activity of Prdx6, such that, higher the percentage inhibition of HRP oxidation by another peroxidase, greater is the catalytic efficiency of that peroxidase. Inset. HRP alone (10 μM, black), HRP (10 μM) followed by addition of 5 μM hydrogen peroxide (red), HRP (10 μM) followed by hydrogen peroxide (5 μM) mixed with 5 μM reduced Prdx6 (blue) or 5 μM oxidised Prdx6 (green); the spectrum was scanned 10 s later.

As seen in Fig. 2, the peroxidase activity of reduced and oxidised Prdx6 was examined using HRP competitive assay^22^. In this assay, estimation of percentage inhibition of hydrogen peroxide-mediated HRP oxidation due to the presence of another anti-oxidant enzyme competing for peroxide substrate, allows assessment of catalytic efficiency of the competing peroxidase. As expected, oxidised Prdx6 due to conversion of reactive thiol to sulfenic acid, was found to be inefficient in competing against HRP, while reduced Prdx6 was found to be highly active showing approx. 80% HRP inhibition. The abridged efficiency of oxidised Prdx6 to block HRP/H_2_O_2_ reaction clearly indicates loss of peroxidase function.

### Conformation of the oxidised and reduced Prdx6 is different

**Figure 3:**
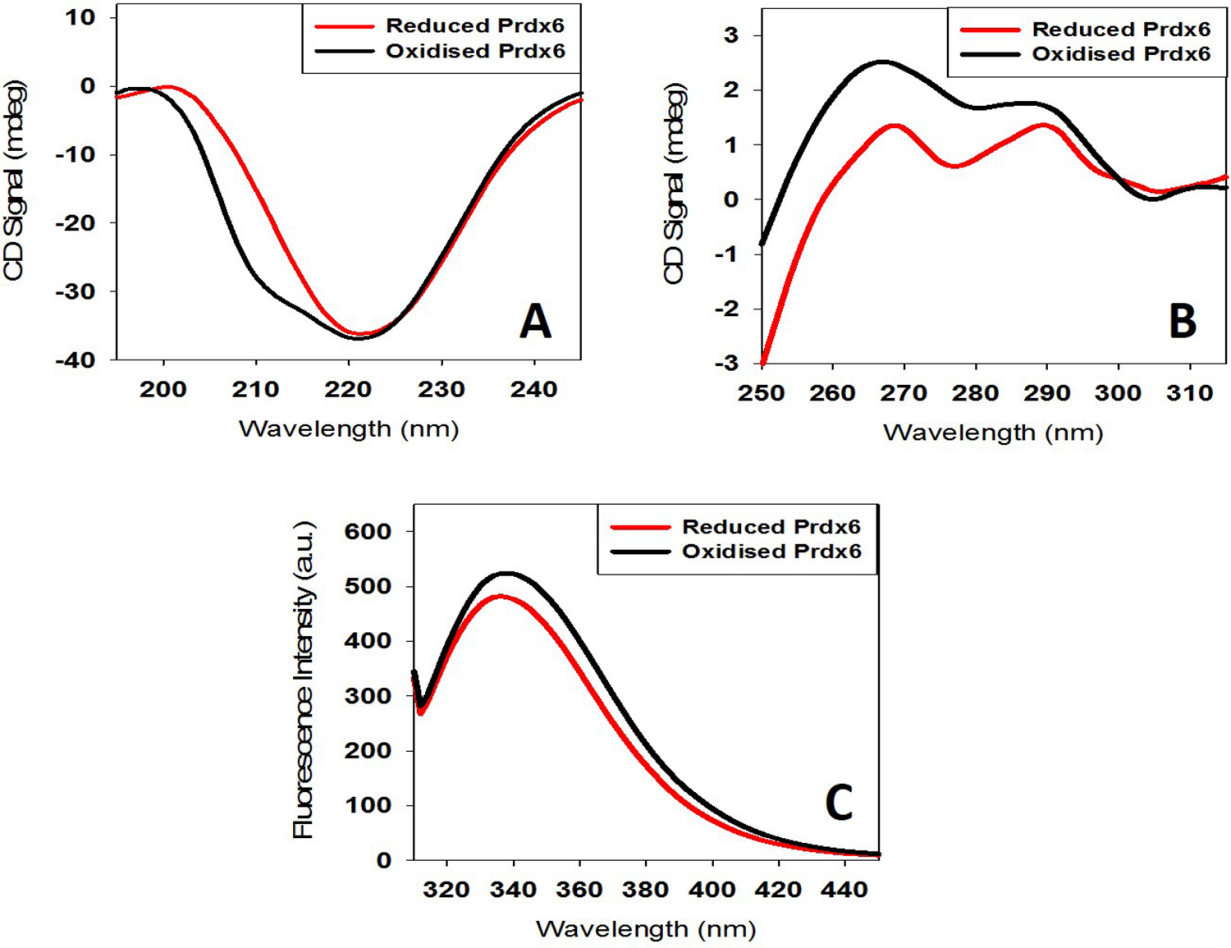
Prdx6 Structure Determination at Different Redox State. The secondary and tertiary structure of Prdx6 at different redox state is determined using (**A**) far-UV CD, (**B**) near-UV CD, and (**C**) tryptophan fluorescence measurements of reduced and oxidised Prdx6. The data is plotted using Sigma Plot 10.0 (Systat Software, Inc., San Jose California, USA, www.systatsoftware.com).

The conformational alterations upon oxidation and reduction of Prdx6 are assessed using several spectroscopic techniques including far-UV CD, near-UV CD and Trp fluorescence (Fig. 3). Far-UV CD spectrum of a protein arises primarily from the spatial arrangements of amide groups such that the negative bands at (i) 208 and 222 nm represents α-helical structures, (ii) 216 nm represents β-sheets, and (iii) 200 nm represents random coils^23^. In this case, we observed increase in the secondary structure of the oxidised protein as compared to the reduced form, and both the proteins to be predominantly α-helical (Fig. 3A). Furthermore, on estimating the percent secondary structural changes in terms of each secondary structural content (α-helix, β-sheet, turns and random coils), we noticed gain in α-helical and β-sheet content with loss of turns and random coil in oxidised Prdx6 (See Table 2). In the near-UV region, as seen in Fig. 3B, Prdx6 displays two prominent peaks at 270 nm and 290 nm, arising from its 12 Phe and 3 Trp residues, respectively. While comparing the near-UV CD spectra for reduced and oxidised Prdx6, we observed a sharp decrease in the Phe peak but only a very small change in Trp peak. We further probed the alteration in tertiary interactions by measuring Trp fluorescence (See Fig. 3C), and observed (a) a slight loss of Trp fluorescence intensity and (b) blue shift (~  2 nm) of reduced Prdx6 in contrast with oxidised Prdx6.

**Table 2:** Secondary Structure Content of Reduced and Oxidized Prdx6 as Measured by Far-UV CD Analysis.

	Alpha helix (%)	Beta sheet (%)	Turns (%)	Random coil (%)
Reduced Prdx6	26.3	23.8	22.7	27.2
Oxidised Prdx6	27.2	25.3	21.6	25.9

### Reduced and oxidised Prdx6 exhibit no significant difference in thermal stability

**Figure 4:**
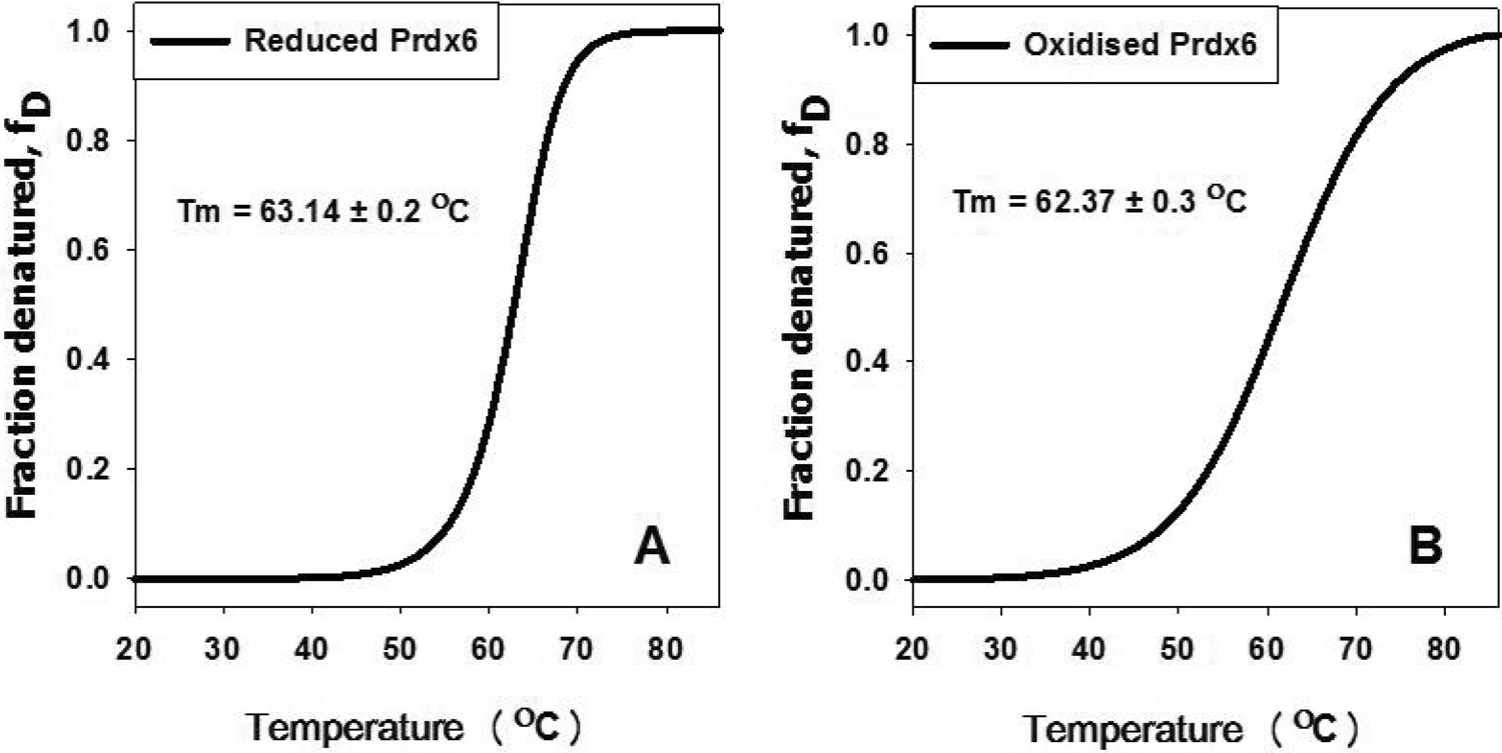
Thermal Stability Measurements of Prdx6 at Different Redox States. The plot shows thermal denaturation profile of (**A**) reduced and (**B**) oxidised Prdx6 as a function of variable temperature. All spectra are mean of three independent experiments, plotted using Sigma Plot 10.0 (Systat Software, Inc., San Jose California, USA, www.systatsoftware.com) and analysed for Tm (midpoint of denaturation) using Origin 7.0 (OriginLab Corporation, Northampton, MA, USA, www.originlab.com).

To investigate for the effect of change in redox status of peroxidatic cysteine on the thermodynamic stability of Prdx6, we carried out heat-induced denaturation study of oxidised and reduced Prdx6 by monitoring the changes in ellipticity at 222 nm as a function of temperature. The change in ellipticity obtained in such a manner was normalized to fraction denatured (f_D_) (Fig. 4). The transition curves obtained from the thermal-induced denaturation were analysed for Tm and ΔHm. We observed that thermodynamic parameters of oxidised and reduced Prdx6 are not significantly different.

### Oligomeric state of reduced and oxidised Prdx6

**Figure 5:**
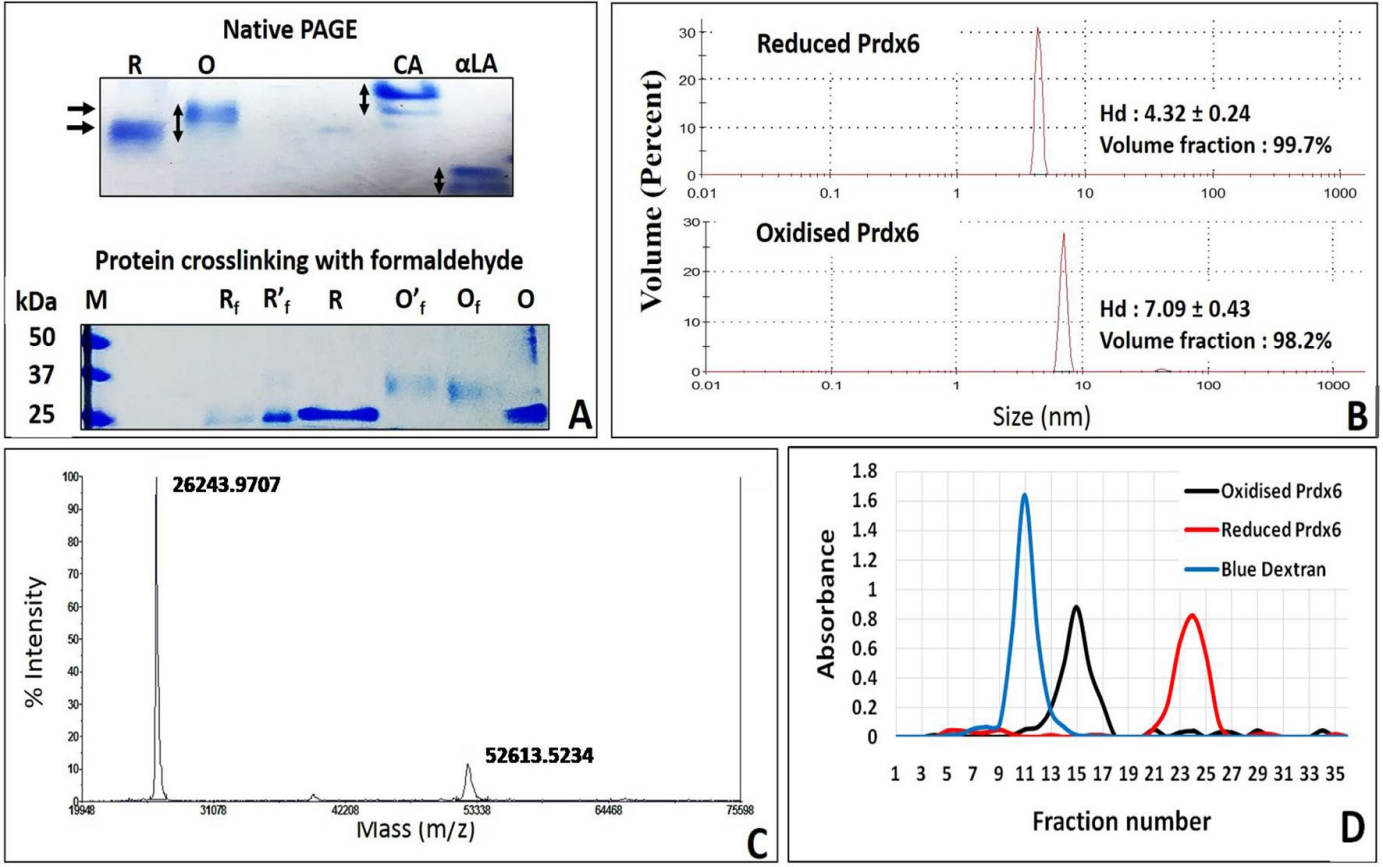
Determining Oligomeric Status of Prdx6 in Reduced and Oxidised State Using Electrophoretic, Spectroscopic and Chromatographic tools. (**A**) Native-PAGE (8%T/0.27%C) (top) and Protein/formaldehyde crosslinking followed by 12% SDS-PAGE (bottom) with reduced and oxidised Prdx6, (**B**) Hydrodynamic diameter (H_d_) measurement by analysing size distribution by volume plot of reduced and oxidised Prdx6 using DLS, (**C**) Intact mass spectroscopic analysis of reduced Prdx6, and (**D**) Elution profile of Blue Dextran (monitored at 600 nm), reduced and oxidised Prdx6 (monitored at 280 nm) on sephadex G-75 column. {Note. R: Reduced Prdx6; O: Oxidised Prdx6; R_f_: Reduced Prdx6 crosslinked with 0.6% formaldehyde; R′_f_: Reduced Prdx6 crosslinked with 1.2% formaldehyde; O_f_: Oxidised Prdx6 crosslinked with 0.6% formaldehyde; O′_f_: Oxidised Prdx6 crosslinked with 1.2% formaldehyde; CA, Carbonic Anhydrase (monomer: 25KDa); αLA, α-lactalbumin (monomer: 14KDa)}.

To investigate the quaternary structure of reduced and oxidised Prdx6, we employed various electrophoretic and spectroscopic methods including native gel electrophoresis, formaldehyde cross-linking, DLS, and mass spectroscopy, (See Fig. 5). The gel electrophoresis of reduced and oxidised Prdx6 in their native state (non-reducing, non-denaturing conditions) clearly displayed bands with different electrophoretic mobility. As seen in Fig. 5A, oxidised Prdx6 was found to have less electrophoretic mobility than reduced Prdx6. The shift in electrophoretic mobility between these two Prdx6′s was found comparable to that of the bands representing monomer and dimeric forms of control proteins, carbonic anhydrase (monomer ~ 30 kDa), and alpha-lactalbumin (monomer ~ 14 kDa). The control proteins used here are well known to exist as both monomer and dimer in their native state^30,31^. The effect of change in redox status on the multimeric status of Prdx6 is also assessed with the help of cross-linking reagent formaldehyde followed by SDS-PAGE gels (Fig. 5A). While reduced Prdx6 displayed no change in its electrophoretic mobility from that of the denatured monomeric protein (molecular weight ~ 26 kDa), oxidised Prdx6 exhibited reduced mobility showing apparent cross-linking with formaldehyde. Our observations are in agreement with earlier cross-linking studies showing EDC/sulfo-NHS crosslinked reduced Prdx6 to be 100% monomeric^13^.

To further evaluate the oligomeric nature of Prdx6, we next performed DLS measurements for reduced and oxidised human Prdx6. DLS is an important tool for analysing the H_d_ and hence multimeric status of the protein. Figure 5B shows the size distribution by volume plot of reduced and oxidised Prdx6. The reduced Prdx6 was found to have H_d_ of 4.32 nm (corresponding to that of a protein with molecular weight 25 kDa) while the oxidised Prdx6 had H_d_ of 7.09 nm (Molecular weight ~ 50 kDa).

The monomeric nature of reduced Prdx6 was confirmed using intact MS and size exclusion chromatography (See Fig. 5C,D).The MS data showed reduced Prdx6 to have a large population corresponding to monomeric (~  26 kDa) protein with a small percentage of dimeric species (~ 52 kDa) (See Fig. 5C). The latter could be because of auto-oxidation of some fraction of reduced Prdx6 by environmental oxygen due to sample processing methodology or incubation time. Size exclusion chromatography, also known as molecular sieve chromatography or gel chromatography is a protein separation technique which is frequently used to analyse/compare the oligomeric status of proteins in their native state, as the elution volume of a molecule on the gel chromatographic column is inversely proportional to the size of the molecule. Figure 5D shows elution profiles (absorbance versus elution volume) of Blue Dextran, Oxidised and Reduced Prdx6 on sephadex G-75 column. As can be seen from the figure, all these components eluted from the column in the form of a single symmetrical peak. Elution volume of the blue coloured fractions of blue dextran monitored at 600 nm represented void volume (11 ml) of the column. The protein, oxidised and reduced Prdx6, when monitored at 280 nm, eluted after the blue dextran peak with an elution volume of 15 ml and 24 ml, respectively. This clearly depicts that molecular weight of oxidised Prdx6 lies between blue dextran and reduced Prdx6. This observation is consistent with our data from protein cross-linking, native PAGE, DLS, MS experiments, which shows reduced Prdx6 to be a monomer of molecular weight 25 kDa and oxidised Prdx6 as the dimer of approximately 50 kDa.

## Discussion

The effect of peroxidatic Cys residue’s redox state on peroxidase activity of human Prdx6 is analysed using HRP/H_2_O_2_ competitive assay. This assay was successfully employed earlier to determine catalytic efficiency of peroxidase activity of rat Prdx6^21^. The main advantage of HRP/H_2_O_2_ competitive assay in comparison with existing assays (as described by Nelson and Parsonage) is its selectivity to reactive protein-cysteine residue which allows peroxidase activity measurement of Prdx6 exclusively in its reduced and oxidised state without usage of any secondary electron donors or additives^21,22^. Our in vitro peroxidase activity measurements via HRP competitive kinetics^22^ revealed that reduced Prdx6 is active (with more than 70% inhibition of HRP peroxidation) while oxidised form is inactive (See Fig. 2).Our observation is in congruence with the typical redox state dependent functional behaviour of peroxiredoxin family proteins^32–34^.

Typical Prdx’s (Prdx1-4) and atypical Prdx (Prdx5) have been identified to show decamer/dimer and monomer/dimer transition, respectively, upon oxidation of their peroxidatic cysteine^8,9,33,35^. However, in case of Prdx6 the behaviour is completely unanswered^33^. While, mass analysis of Prdx6 extracts/recombinant Prdx6 reveals that the protein exists in monomer–dimer equilibrium^14–17^, the exact redox state of the samples was not analysed in these studies. Available reports on the native state crystal structure of reduced and oxidised Prdx6 are also conflicting^10,11^ and have several limitations or deficiencies^7,12,13^. These observations further engaged our curiosity to understand the structural foreplay underlying the peroxidase catalytic cycle. First of all we have investigated the oligomeric nature of the oxidized and reduced proteins. Our investigations using electrophoretic methods, DLS, mass spectroscopy, and size exclusion chromatography revealed reduced Prdx6 to be monomeric (Mw: 25 kDa, H_d_: 4.32) and oxidised Prdx6 to be dimeric (Mw: 50 kDa, H_d_: 7.09 nm) in nature. Such transitions are not uncommon for peroxiredoxin family proteins, which are known to alter their conformation drastically and control their peroxidase activity by changing oxidation status of active site Cys residue^6,8,9^. It is to be noted here that such dimerization behaviour in case of Prdx6 does not involve disulfide formation between two monomers because unlike the 2-Cys Prdx’s where the catalytic Cys residues are on the surface and available to form disulfides, the peroxidatic Cys 47 of Prdx6 is located at the bottom of a narrow pocket with diameter and depth ~ 4 Å and ~ 7 Å respectively, thereby being unable to form a disulfide bond. Crystal structure report also support this premise^8,11^. Moreover, it is believed that upon dimerization, the substrate accessibility of peroxidatic cysteine group is blocked because of the entry of P191 residue of one monomer into the catalytic pocket of another monomer, suggesting the process of dimer-monomer transition necessary for successful completion of the peroxidase catalytic cycle^11^. Thus the dimerization mechanism (or enzyme inactivation) in case of Prdx6 is different from those of typical 2-Prdx’s wherein disulfide-induced homodimerization is considered to be the emblematic signatures^8,33^.

Absence of disulfide-induced dimerization in Prdx6 advocates that dimer formation might involve other structural alterations. We have therefore, intentionally examined the structural alterations that could lead to dimer formation. For this we have used several spectroscopic tools to analyse the conformational variations between the oxidised and reduced Prdx6. It is evident in Fig. 3A and Table 2 that there is creation of new secondary structural components, exemplified by the increase in alpha helix and beta sheets content with concomitant decrease in the random coil in the oxidised protein. These new structural creations also appear to affect the beta turn components (see Table 2). Furthermore, the new addition of secondary structure components in turn brings about changes in the tertiary structure of the oxidised form (as evident from the increased near UV, and Trp fluorescent measurements) (Fig. 3B and C). Such structural alterations might affect the exposure of the hydrophobic groups in the protein, which might be responsible to initiate hydrophobic contacts-mediated dimerization upon oxidation. Consistent with this observation, crystal structure analysis of human Prdx6 disclosed that dimer formation requires two important hydrophobic residues, L145 and L148^11^. Thus the overall structural alterations brought about by oxidation results in the exposure of important residues to initiate hydrophobic contact formation. Taken together, the results led us to conclude that in contrast to dimerization of other Prdx’s which involves disulfide bridges, oxidised Prdx6 forms dimer via a different mechanism.

The changes in the conformation of the two states might have affected the thermodynamic stability of the two different states. It is seen in Fig. 4 that though the co-operative nature of the transition curves are somewhat dissimilar, the analysed Tm values of the proteins are not significantly altered indicating that in vitro thermodynamic stability and hence the cellular proteo-stability of the oxidised and reduced forms of Prdx6 are similar. Perhaps the increase in both the alpha helix and beta sheets along with the concomitant loss in the beta turn might account for the observed structural compensation leading to no change in overall thermodynamic stability of the two protein forms. Thus it appears that redox changes in Prdx6 occur at the cost of structural stability.

## Summary

The study confirms that reduced Prdx6 is monomeric while oxidised form is dimeric. Our findings also indicate that the change in the redox status of Prdx6 is associated with certain structural transitions at both secondary and tertiary level. These transitions appear to have a definitive role in determining the subunit association propensity of Prdx6 which in turn helps to modulate its peroxidase activity. The peroxidase catalytic cycle of Prdx6 constitutes the majority of protein in 2 different states—(a) reduced form, and (b) oxidised form. In the peroxidase catalytic cycle, the first step involves monomeric reduced Prdx6 encountering a peroxide substrate, reducing it into water and getting self-oxidised. Thus formed, sulfenic form of Prdx6, however, has a different structure (than reduced Prdx6) that favours dimerization, consequently blocking the active site Cys and thereby preventing its direct reduction by various soluble antioxidants like thioredoxin / NADPH / glutathione.

## Supplementary information

Supplementary Information.
