# Supplementary material for: Structural basis of peroxidase catalytic cycle of human Prdx6
Source: Sci Rep. 2020 Oct 15;10:17416. doi: 10.1038/s41598-020-74052-6 (PMC7566464; doi:10.1038/s41598-020-74052-6)

SUPPLEMENTARY INFORMATION

Structural basis of Peroxidase Catalytic Cycle of human Prdx6

Rimpy Kaur Chowhan[1], Hamidur Rahaman[2] and Laishram Rajendrakumar Singh[1,*]
[1] *Dr. B.R. Ambedkar Center for Biomedical Research, University of Delhi, Delhi 110007, India*
[2]Department of Biotechnology, Manipur University, Imphal, India 795003

[*]**Corresponding author**: Laishram R. Singh. Tel: +91-9811630757; E-mail: lairksingh@gmail.com.

Running title: Structural analysis of reduced and oxidised human Prdx6

Below are the full-length gels and blots corresponding to the figures in the main manuscript file. For complete legends corresponding to each gel, please refer the main manuscript.

Figure S1. Full-length Gel Image corresponding to Figure 1A. Here Lane 6 to 10 correspond to the legends described as M, UI, Sup, and P, respectively in figure 1A

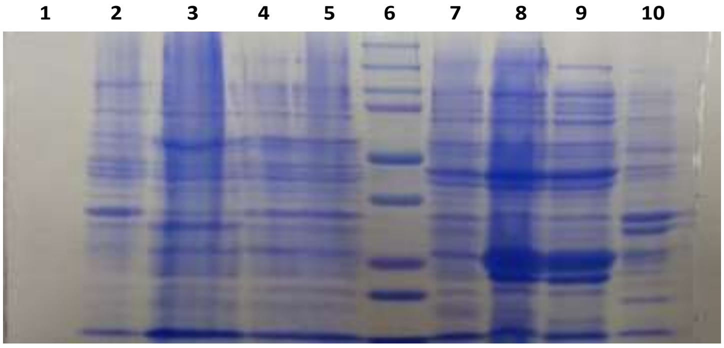

Figure S2. Full-length Gel Image corresponding to Figure 1B. Here lane 1-10 corresponds to the legend described as M, Sup, F, W1, W2, W3, E1, E2, E3, and E4, respectively in figure 1B

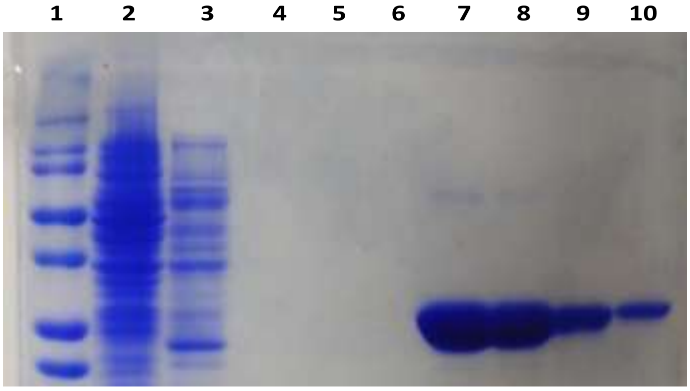

Figure S3. Full-length Gel Image corresponding to Figure 1C. Here Lane 7, 9 and 10 correspond to the legends described as M, ED1, and ED2, respectively in figure 1C.

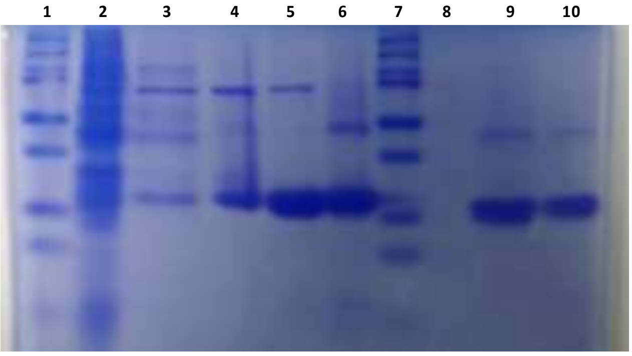

Figure S4. Full-length Immunoblot Image corresponding to Figure 1D

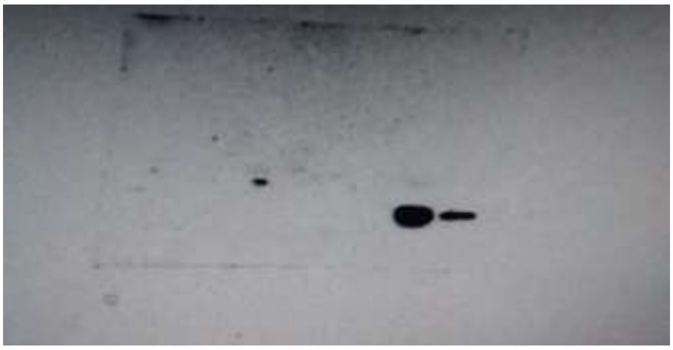

Figure S5. Full-length Gel Image corresponding to Figure 5A (gel showing Native PAGE). Here Lane 1 is marker, Lane 3, 4, 8, and 9 is described as R, O, CA, αLA in the legends in figure 5A (for native PAGE). In lane 10, we have loaded Bovine serum albumin as positive control to see its monomer and multimers. The point was to show, that the difference in the electrophoretic mobility of a monomer and dimer is different for every protein.

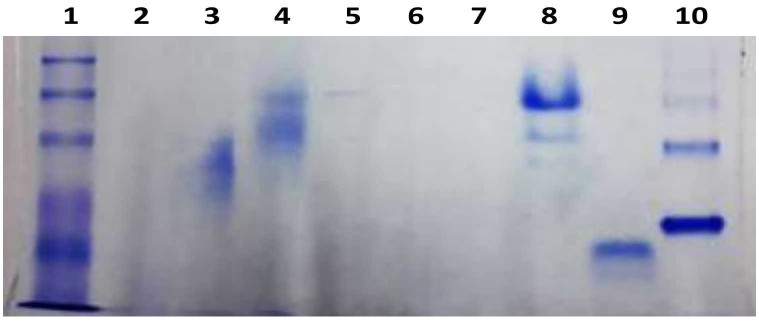

Figure S6. Full-length Gel Image corresponding to Figure 5A (gel showing formaldehyde crosslinking followed by SDS- PAGE). Here Lane 1, 3 to 8 correspond to the legends described as M, R_f, R'_f, R, O_f, O'_f, O, respectively in figure 5A. The protein sample crosslinked with 5% formaldehyde, loaded in lane 9, aggregated and has been excluded from the image in manuscript to avoid discussing failed standardization condition.

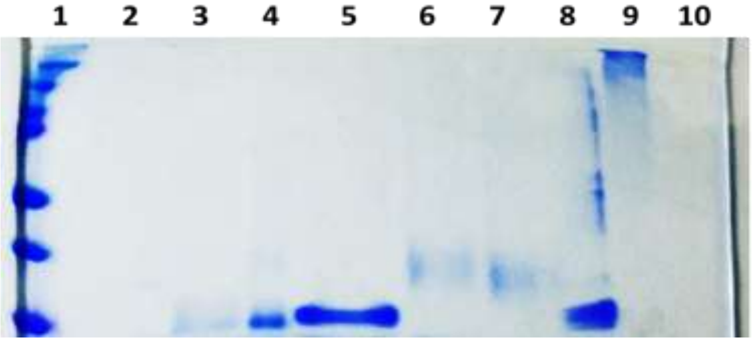

Supplement: Supplementary file 1 — Supplementary Information. [file 41598_2020_74052_MOESM1_ESM.pdf]
